# Six‐year multi‐centre, observational, post‐marketing surveillance of the safety of the HPV‐16/18 AS04‐adjuvanted vaccine in women aged 10–25 years in Korea

**DOI:** 10.1002/pds.4175

**Published:** 2017-03-07

**Authors:** Chul‐Jung Kim, Rok Song, Jing Chen, Fernanda Tavares Da Silva, Kusuma B. Gopala, Joon Hyung Kim, Dan Bi, Jong Sup Park

**Affiliations:** ^1^ Department of Obstetrics and Gynecology, College of Medicine Konyang University Daejeon Korea; ^2^ GSK Vaccines Seoul Korea; ^3^ GSK Pte Ltd Singapore Singapore; ^4^ GSK Vaccines Wavre Belgium; ^5^ GSK Pharmaceuticals Ltd Bangalore India; ^6^ Department of Obstetrics and Gynecology, Seoul St. Mary's Hospital The Catholic University of Korea Seoul Korea

**Keywords:** Korea, post‐marketing surveillance, safety, HPV‐16/18 AS04‐adjuvanted vaccine

## Abstract

**Purpose:**

To evaluate the safety of HPV‐16/18 AS04‐adjuvanted vaccine when administered as per the PI in Korea.

**Methods:**

A total of 3084 women aged 10–25 years were enrolled in this post‐marketing surveillance from 2008 to 2014. Subjects were invited to receive three doses of the vaccine (0, 1 and 6 months), and participants who received at least one dose were included in the analysis. Adverse events (AEs), adverse drug reactions (ADRs) and serious AEs (SAEs) were recorded after each dose. All AEs, ADRs and SAEs were presented with exact 95% confidence intervals (CI) (NCT01101542).

**Results:**

Injection‐site pain was the most frequent AE and ADR reported by 322 subjects (10.4% [95%CI: 9.4–11.6]); the local pain was transient and lasted 4–7 days in most cases. Dysmenorrhoea and vaginitis were the most common unexpected AEs reported by 30 (1.0% [95%CI: 0.7–1.4]) and 16 subjects (0.7% [95%CI: 0.3–0.8]), respectively. Pain (toe pain, leg pain and body pain [one case each]; foot pain [two cases]) was the most common unexpected ADR reported by five subjects (0.2% [95%CI: 0.1–0.4]). Four subjects reported a single SAE (one case each of exostosis, gastroenteritis, abortion and tonsillitis); none were fatal. All SAEs were assessed as unlikely to be related to vaccination; gastroenteritis, exostosis and tonsillitis resolved during the study period.

**Conclusions:**

This is the first post‐marketing surveillance study in Korea that provides 6‐year safety data for HPV‐16/18 AS04‐adjuvanted vaccine. The vaccine showed an acceptable safety profile and favourable benefit/risk ratio when given to women aged 10–25 years in Korea. © 2017 The Authors. Pharmacoepidemiology & Drug Safety Published by John Wiley & Sons Ltd.

## Introduction

Cervical cancer (CC) is one of the most common cancers worldwide among women, with 527 624 new cases and 265 653 deaths every year.^1^ In 2012, CC has been estimated to be the seventh most common cancer among women in Korea and had the fourth highest age‐standardised incidence rate (9.5 per 100 000) among Eastern Asia countries (Mongolia, North and South Korea, Japan and China).^2^

Persistent infection with an oncogenic genotype of human papillomavirus (HPV) is a necessary cause of CC; although, many HPV infections are resolved naturally without any external intervention.^3^, ^4^ In fact, 99.7% of CC cases worldwide showed the evidence of HPV infection.^3^ HPV‐16 and HPV‐18 are the most common HPV types detected in about 70% of global CC^5^ and 70.3% CC cases in Korea.^2^ HPV‐18 is the most frequent in cervical adenocarcinoma in Korean women (41.6%), followed by HPV‐16 (38.1%), HPV‐45 (3.1%), HPV‐ 33 (2.0%) and, HPV‐31 and HPV‐68 (1.3%).^2^ HPV‐16 is the most frequent in invasive CC (54.4%). Amongst other carcinogenic HPV types, HPV‐33 and HPV‐58 are highly prevalent in invasive cancer and high‐grade cervical neoplasia in Korean women.^2^

There are 21.24 million women over 15 years of age in Korea who are at risk of developing CC.^2^ The prevalence of HPV infection ranges between 10 and 40% in Korean women up to 65 years of age with normal cervical cytology.^2^ Of these, women aged under 25 years show the highest prevalence of HPV infection.

The HPV‐16/18 AS04‐adjuvanted vaccine (*Cervarix™*, manufactured by GSK, Belgium) has been approved in 135 countries worldwide.^6^ The vaccine has been shown to be efficacious in preventing 93.2% of high grade cervical intraepithelial neoplasia (CIN3+) in naive population with an acceptable safety profile and sustained immunogenicity.^7^, ^8^, ^9^, ^10^, ^11^ Post‐licensure surveillance has confirmed the safety profiles of the vaccine in many populations.^12^, ^13^, ^14^, ^15^ The Korean Ministry of Food and Drug Safety (MFDS) approved the use of the HPV‐16/18 AS04‐adjuvanted vaccine in Korea in 2008.^16^ This post‐marketing surveillance (PMS) study was undertaken to evaluate the safety of the HPV‐16/18 AS04‐adjuvanted vaccine when given to women aged 10–25 years in Korea, according to the Korean prescription information (PI).

## Methods

### Study design

This PMS was conducted between July 2008 and July 2014 at 48 centres in Korea (NCT01101542). HPV‐16/18 AS04‐adjuvanted vaccine was administered to women aged 10–25 years according to local PI. Girls and women who received at least one dose of the vaccine during the study period were invited to the study. All participants were included in the total vaccinated cohort (TVC).

### Ethics

The study was conducted in accordance with regulatory requirements and the local rules and regulations (MFDS). Subjects or parents/guardians of subjects provided written informed consent for the collection and handling of personal and safety information before conducting any study‐specific procedures.

### HPV‐16/18 AS04‐adjuvanted vaccine

The HPV‐16/18 AS04‐adjuvanted vaccine was developed and manufactured by GSK Vaccines, Belgium, with each 0.5‐ml vaccine dose containing 20 μg of each HPV type‐specific virus‐like particles (types 16 and 18) composed of the L1 major capsid proteins, adjuvanted with AS04 (50‐μg monophosphoryl lipid A and 500‐μg Al[OH]_3_). The vaccine was applied intramuscularly in the deltoid region of the arm as per the local PI in Korea.

### Safety assessment

Adverse events (AEs) that occurred after the vaccination of each dose were recorded in diary cards and classified as either expected (AEs included in the PI) or unexpected. The intensity of AEs was recorded as: mild (easily tolerated by the subject, causing minimal discomfort and not interfering with the everyday activities), moderate (sufficiently discomforting to interfere with normal everyday activities; fever >38 to ≤39 °C) or severe (prevented normal, everyday activities; fever >39 °C).

Causal relationships of AE were categorised as; certain, probable, possible, unlikely, conditional and unassessable (as defined in Supplementary Table 1). An adverse drug reaction (ADR) was any AE of which causal relationships were categorised as certain, probable, possible, conditional and unassessable, as assessed by the investigator. The definitions of causality assessment were based on the guidance by Korean authorities. Adverse events categorised as unlikely were not classified as ADRs.

**Table 1:** Occurrence, duration and investigator's causality assessment of injection‐site pain following each dose of vaccine

Days	Post dose 1 (*N* = 240) *n* (%)	Post dose 2 (*N* = 98) *n* (%)	Post dose 3 (*N* = 68) *n* (%)
1	27 (11.1)	12 (12.2)	6 (9.0)
2	61 (24.9)	34 (34.7)	21 (31.3)
3	64 (26.1)	23 (23.5)	17 (25.4)
4–7	74 (30.2)	25 (25.5)	20 (29.9)
8–15	14 (5.7)	3 (3.1)	2 (3.0)
16–30	5 (2.0)	0(0)	1 (1.5)
>30	—	1 (1.0)	—
Duration unknown	5 (2.0)	—	—
Causality assessment to the vaccine			
Certainly	177 (70.8)	87 (88.8)	62 (91.2)
Probably	49 (19.6)	8 (8.2)	6 (8.8)
Possibly	24 (9.6)	3 (3.1)	—

*N*: Total number of subjects, *n*(%): number (percentage) of cases with injection‐site pain.

Serious AEs (SAEs), pregnancies and their outcome, concomitant medication/vaccination, contraindication to vaccination and other medical emergencies were recorded throughout the study until one month after the last dose of vaccine.

### Statistical analysis

Statistical analyses were performed on the TVC. Adverse events and SAEs stratified by expected and unexpected based on the WHOART dictionary were analysed with exact 95% confidence intervals (CI). All statistical analyses were conducted on the Statistical Analysis Systems, (Cary, NC, USA).

## Results

### Baseline characteristics

A total of 3084 women, enrolled during the 6‐year surveillance period, were included in the TVC for safety analyses; 2338 (75.8%) subjects received three doses of the vaccine; 746 (24.2%) subjects were incompletely vaccinated: 455 (14.7%) subjects received two doses and 291 (9.5%) subjects received only one dose of the vaccine.

The mean age at first dose was 20.5 years (standard deviation: 3.8). The vast majority of subjects [3077 (99.8%)] were of Korean ethnicity and 7 (0.2%) subjects were non‐Korean (four Vietnamese, two Chinese and one subject of unknown ethnicity). Nine‐hundred forty‐two (30.5%) subjects had a pre‐existing medical history including infections and infestations other than HPV [441 (14.3%)], reproductive system and breast disorders [179 (5.8%)] and skin and subcutaneous tissue disorders [135 (4.4%)]. Concomitant medication including antibiotics [176 (5.7%)] and antipyretics [146 (4.7%)] was taken by 369 (12.0%) subjects during the post‐vaccination periods. There were 65 (2.1%) subjects who received other vaccines such as Influenza vaccines and Hepatitis A and B vaccines concomitantly with the study vaccine.

### Safety results

All AEs, ADRs and SAEs were reported after each dose. Overall 632 subjects (20.5%) experienced 1154 AEs. Three hundred and twenty‐two subjects (10.4%) reported injection‐site pain, the most frequently reported AE and ADR. All other AEs and ADRs were reported by ≤1.5 and 1.2% of subjects, respectively. There were more AEs reported after dose 1 (667 AEs reported by 425 subjects [18.2% {95%CI: 16.6–19.8}]), than after dose 2 (277 AEs reported by 213 subjects [9.9% {95%CI: 8.6–11.2}]) and dose 3 (207 AEs reported by 156 subjects [7.4% {95%CI: 6.4–8.6}]).

A total of 479 subjects (15.5% [95%CI: 14.3–16.9]) reported at least one expected AE (852 cases of AE in total) that resolved/recovered. Two subjects (0.1% [95%CI: 0.0–0.2]) each reported one expected AE (two cases) for which the outcome of the AE was unknown. The expected AEs reported by participants were more frequently mild (reported by 409 subjects, 13.3% [95%CI: 12.1–14.5]) than moderate (reported by 121 subjects, 3.9% [95%CI: 3.3–4.7]) or severe (reported by nine subjects, 0.3% [95%CI: 0.1–0.6]) (intensity was missing for one subject who reported at least one expected AE).

Injection‐site pain was the most common expected AE and ADR. In most cases, the pain lasted for between 4 and 7 days, irrespective of dose (Table 1). At least 70% of the cases of injection‐site pain were judged to be certainly related to vaccination (Table 2).

**Table 2:** Most frequent adverse events and adverse drug reactions classified as expected and unexpected, during the post‐vaccination follow‐up period

		Adverse event					Adverse drug reaction	
	Most frequent symptom		*n* (%)	95%CI (LL–UL)	Most frequent symptom		*n* (%)	95%CI (LL–UL)
Expected	Injection‐site pain	Overall	322 (10.4)	9.4–11.6	Injection‐site pain	Overall	322 (10.4)	9.4–11.6
		Post dose 1	240 (10.3)	9.1–11.6		Post dose 1	240 (10.3)	9.1–11.6
		Post dose 2	98 (4.5)	3.7–5.5		Post dose 2	98 (4.5)	3.7–5.5
		Post dose 3	68 (3.2)	2.5–4.1		Post dose 3	68 (3.2)	2.5–4.1
Unexpected	Dysmenorrhoea	Overall	30 (1.0)	0.7–1.4	Pain	Overall	5 (0.2)	0.1–0.4
		Post dose 1	16 (0.7)	0.4–1.1		Post dose 1	4 (0.2)	0.0–0.4
		Post dose 2	15 (0.7)	0.4–1.1		Post dose 2	2 (0.1)	0.0–0.3
		Post dose 3	2 (0.1)	0.0–0.3		Post dose 3	—	—
	Vaginitis	Overall	16 (0.5)	0.3–0.8				
		Post dose 1	12 (0.5)	0.3–0.9				
		Post dose 2	3 (0.1)	0.0–0.4				
		Post dose 3	3 (0.1)	0.0–0.4				

*n* (%): number (percentage) of subjects in the specified category; LL and UL: lower limit and upper limit of 95% confidence interval (CI), respectively.

A total of 294 unexpected AEs were reported by 226 subjects (7.3% [95%CI: 6.4–8.3]) with 40 unexpected ADRs experienced by 26 subjects (0.8% [95%CI: 0.6–1.2]). Overall, 191 subjects (6.2% [95%CI: 5.4–7.1]) reported at least one unexpected AE (246 cases) that resolved/recovered by the end of the PMS period. Dysmenorrhoea and vaginitis were the most common unexpected AEs. Pain was assessed by the investigators as the most common unexpected ADR, with two cases of foot pain and one case each of toe pain, leg pain and body pain (Table 2). All other unexpected AEs and ADRs were reported by ≤0.2 and ≤0.1% subjects, respectively. Table 3 shows the causality assessments for the unexpected AEs/ADR and the vaccination, as per investigator's assessment. The majority of dysmenorrhoea cases were considered to be unlikely to be related to vaccination; one case was conditionally related. Amongst all cases of vaginitis, one case was assessed as having an unlikely relationship to vaccination, and one case was judged to be certainly related to vaccination. Majority of unexpected AEs reported (in 191 subjects) had recovered/resolved by the end of PMS period.

**Table 3:** Investigator's causality assessment of the most common unexpected adverse events

	Post dose 1		Post dose 2		Post dose 3	
	Dysmenorrhoea (*N* = 16)	Vaginitis (*N* = 12)	Dysmenorrhoea (*N* = 16)	Vaginitis (*N* = 3)	Dysmenorrhoea (*N* = 2)	Vaginitis (*N* = 3)
	*n* (%)	*n* (%)	*n* (%)	*n* (%)	*n* (%)	*n* (%)
Certain	—	—	—	—	—	1 (33.3)
Probably	—	—	—	—	—	—
Possibly	—	—	—	—	—	—
Unlikely	15 (93.8)	12 (100)	16 (100)	3 (100)	2 (100)	2 (66.7)
Conditional	1 (6.3)	—	—	—	—	—

*N*: Total number of subjects, *n*: number of cases with injection‐site pain.

Four SAEs (one case each of serious unexpected AEs: exostosis, gastroenteritis, abortion; and serious expected AE: tonsillitis) were reported by four subjects during the entire study, and none were fatal. No SAEs were reported after the last dose of HPV‐16/18 AS04‐adjuvanted vaccine. All SAEs were assessed as unlikely to be related to the vaccine and the exostosis, gastroenteritis and tonsillitis resolved by the end of the PMS period. No serious ADR was reported.

## Discussion

Cervical cancer is a major global health concern for women, including women in Korea.^2^ Recent studies have shown an increased occurrence of HPV infection among Korean adolescents due to a younger age of sexual debut.^17^, ^18^, ^19^ Prophylactic vaccination against HPV provides protection against CC caused by the most commonly found oncogenic HPV types^20^ and is a major public health breakthrough.^21^

HPV‐16/18 AS04‐adjuvanted vaccine elicited high and sustained antibody levels against HPV‐16 and HPV‐18 with acceptable safety profiles in two clinical trials among Korean women aged 10–14 and 15–25 years, respectively.^22^, ^23^ In other clinical trials and surveillance studies conducted in different countries such as Japan, the Netherlands and United Kingdom^7^, ^8^, ^9^, ^10^, ^11^, ^12^, ^13^, ^14^, ^15^, ^24^, ^25^, ^26^, ^27^, ^28^, ^29^, transient injection‐site pain lasting for less than 5 days ^30^ was consistently the most commonly occurring AE. The results of the present study now provide further evidence that the safety profile of the HPV‐16/18 AS04‐adjuvanted vaccine is similar when administered to women in Korea.

This PMS evaluated the safety of HPV‐16/18 AS04‐adjuvanted vaccine given to 3084 women in Korea as per routine clinical practice. The most common expected AE and ADR were a local injection‐site reaction, i.e. injection‐site pain. While the pain was short lived as expected with any intramuscular administration of a vaccine, it was also less frequent at the administration of the second and third doses of the vaccine. Systemic effect of the vaccination was acceptable and most of the expected AEs reported were mild in intensity. The most frequently reported unexpected AEs were dysmenorrhoea and vaginitis. However, most cases were considered unlikely to be related to vaccination. Pain (toe, leg, foot and body) was the most frequently reported unexpected ADR.

Over 70 000 doses of the HPV‐16/18 AS04‐adjuvanted vaccine were administered through clinical trials prior to licensing and over 59 million doses have been distributed globally since its launch. The vaccine showed an acceptable safety profile during the clinical development and in observational studies or PMS.^12^, ^13^, ^14^, ^15^ In June 2013, following the media report of cases resembling Complex Regional Pain Syndrome (CRPS) in Japan after 8 million doses of HPV vaccines were administered, the Japanese Ministry of Health, Labour and Welfare (MHLW) suspended the proactive recommendation of HPV vaccines although no causal link was established by the Vaccine Adverse Reactions Review Committee.^31^ Subsequently, the uptake of HPV vaccines in Japan plunged.^32^ The suspension of the MHLW recommendation also negatively impacted the confidence on the safety of HPV vaccines in other countries.^33^ The World Health Organisation (WHO) Global Advisory Committee for Vaccine Safety regularly reviews the evidence on the safety of HPV vaccines including the HPV‐16/18 AS04‐adjuvanted vaccine. In its position paper on HPV vaccines published in October 2014, the WHO confirmed that the HPV vaccines continue to have an excellent safety profile.^34^ In July 2015, the European Medicines Agency's Pharmacovigilance Risk Assessment Committee (PRAC) started the review of evidence surrounding CRPS and postural orthostatic tachycardia syndrome in young women receiving HPV vaccines (including data from Japan). It confirmed that the available evidence did not support that the HPV vaccines cause CRPS or postural orthostatic tachycardia syndrome.^35^ Based on the PRAC assessment, the European Commission made the decision in November 2015 that there is no reason to change the way the current available HPV vaccines are used or amend the current product information, and agrees that the benefits of HPV vaccines continue to outweigh the risks.^35^

This 6‐year PMS study was conducted at multiple hospitals and clinics in Korea after the approval of the HPV16/18 AS04‐adjuvanted vaccine by the Korean MFDS. More than 3000 girls and women were observed following vaccination with the HPV16/18 AS04‐adjuvanted vaccine as part of the routine clinical practice, which significantly increased the safety data of the vaccine in the Korean population. Findings of this PMS study were consistent with observations during the development of the vaccine as well as other PMS studies conducted in various countries. We conclude that the HPV‐16/18 AS04‐adjuvanted vaccine has an acceptable safety profile when given to women aged 10–25 years in Korea according to the local PI. Potential adverse reactions following vaccination will be continued to be monitored through the routine safety reporting channels.

## Conflict of Interest

GlaxoSmithKline Biologicals SA was involved in all stages of the study conduct and analysis; and also took charge of all costs associated with developing and publishing the manuscript. Rok Song, Jing Chen, Fernanda Tavares Da Silva, Kusuma B Gopala, Joon Hyung Kim and Dan Bi are employees of the GSK group of companies. Joon Hyung Kim, Jing Chen and Rok Song received stock/stock options from GSK Vaccines. Dan Bi and Fernanda Tavares Da Silva own restricted shares as employee's benefits. Chul‐Jung Kim and Jong Sup Park have nothing to disclose.

**Key Points**

- This is the first PMS study in Korea that provides 6‐year safety data for *HPV‐16/18 AS04‐ adjuvanted vaccine* administration in women according to the prescription information.
- The vaccine showed an acceptable safety profile and favourable benefit/risk ratio when given to women aged 10–25 years in Korea. Injection‐site pain was the most common reaction to vaccination.
- Four subjects reported four SAEs (one case each of exostosis, gastroenteritis, abortion and tonsillitis) during the study and were unlikely to be caused by the vaccination; none of the SAEs were fatal.

### Trademark Statement

*Cervarix* is a registered trademark of the GSK group of companies.

## Author Contributions

All authors participated in the design or implementation or analysis, and interpretation of the study and in the development of this manuscript. All authors had full access to the data and gave final approval before submission. The corresponding author was responsible for submission of the publication.

## Supporting information


**Supplementary Table 1.** Definitions of types of causalities as per guidance by Korean authorities.
