# Supplementary material for: Six‐year multi‐centre, observational, post‐marketing surveillance of the safety of the HPV‐16/18 AS04‐adjuvanted vaccine in women aged 10–25 years in Korea
Source: Pharmacoepidemiol Drug Saf. 2017 Mar 7;26(7):837–42. doi: 10.1002/pds.4175 (PMC5516175; doi:10.1002/pds.4175)
Supplement: Supplementary file 1 — Supplementary Table 1. Definitions of types of causalities as per guidance by Korean authorities. [file PDS-26-837-s001.docx]

**Supplementary table 1**. Definitions of types of causalities as per guidance by Korean authorities.

| **Causality** | **Definition** |
| --- | --- |
| Certain | There is a reasonable relationship between the observed reaction and the given vaccine without possible explanation by any concomitant medication or disease |
| Probable | There is an appropriate sequence of vaccination and the observed reaction could probably be explained by any concomitant medication or disease |
| Possible | There is an appropriate sequence of vaccination and the observed symptom could possibly be explained by any concomitant medication or disease. |
| Unlikely | The reported symptom was transient and could be explained reasonably by any concomitant medication or disease |
| Conditional | Additional information was required or the reported case was being reviewed |
| Unassessable | There was insufficient or conflicting data that could not be validated by any means |
